# Dual Modulation of Autophagy and Apoptosis as Anticancer Mechanism of Action of *Khaya grandiofoliola* in Colon Carcinoma Cells

**DOI:** 10.3390/ijms26115247

**Published:** 2025-05-29

**Authors:** Saheed O. Anifowose, Musa K. Oladejo, Abdalrhaman M. Salih, Layali M. Almutairi, Mansour I. Almansour, Badr Al-Dahmash, Mobarak S. Al Mosallam, Ibrahim O. Alanazi, Ahmed Rady

**Affiliations:** 1 Zoology Department, College of Science, King Saud University, P.O. Box 2455, Riyadh 11451, Saudi Arabia; olaidesaeed@hotmail.com (S.O.A.);; 2 Botany and Microbiology Department, College of Science, King Saud University, P.O. Box 2455, Riyadh 11451, Saudi Arabia; 3 Molecular and Cell Biology Laboratory, Prince Naif bin AbdulAziz Health Research Center, College of Dentistry, King Saud University Medical City, King Saud University, Riyadh 11545, Saudi Arabia; 4 Healthy Aging Research Institute, Health Sector, King Abdulaziz City for Science and Technology, Riyadh 11442, Saudi Arabia

**Keywords:** *Khaya grandiofoliola*, anticancer phytochemicals, apoptosis, autophagy, natural products

## Abstract

*Khaya grandiofoliola* (Kh) is a medicinal plant with therapeutic properties. Studies have reported on the general bioactivity and anticancer potentials of the plant, but no investigations have yet investigated its anticancer mechanism of action. This study presents the first examination of the anticancer mechanism of action of the methanolic extract of Kh, alongside phytochemical profiling of its anticancer constituents. We conducted in vitro investigations into the mechanism of action of Kh and performed bioactivity-guided fractionation, with subsequent identification of its anticancer phytochemicals using HPLC and GC-MS, respectively. Kh posed a potent antiproliferative effect against colon carcinoma cells and an antioxidant property at low microgram levels. Furthermore, the treatment of Kh in *Caco-2* cells led to the accumulation of p62 puncta, indicating inhibition of autophagic flux degradation. Kh impacts microtubule, induced G1 arrest, and late apoptosis induction in *Caco-2* cells. Phytochemicals belonging to sesquiterpene alcohols were found most abundant in the Kh bioactive fractions. The identified phytochemicals are potential inducers of apoptosis, autophagy flux inhibition, and G1-phase arrest. Our findings suggest that the anticancer property of Kh is mediated through the dual modulation of autophagy and apoptosis. Further studies are needed to isolate the active compounds responsible for these effects and further elucidate the underlying molecular mechanisms.

## 1. Introduction

Medicinal plants are an excellent repository for bio-prospecting natural products that can be used as drug leads in the drug development process. They are known to house a gamut of intriguing secondary metabolites with therapeutic values [1]. *Khaya grandifoliola* C. DC., henceforth referred to as Kh, belongs to the Meliaceae family. African mahogany is widely used in the trado-medicinal practice in tropical African countries. The plant’s various parts have been reported in the treatment of fever, malaria, gastrointestinal disorders, and inflammatory diseases [2,3]. Human colon adenocarcinoma is the second leading cancer mortality, according to the Globocan 2022 report [4]. Natural products from plants are potent inducers of apoptosis, autophagy, cell cycle arrest, antioxidant effects, and many more cellular processes known to be less functioning in cancer [5]. The phytochemical profile of Kh has been reported for the presence of various bioactive phytochemical classes, including the limonoids, flavonoids, terpenoids, and alkaloids [2,6,7]. Phytochemicals from these classes are known to potently elicit molecular responses in anticancer cellular targets by activating caspases, regulating pro- and anti-apoptotic proteins, disrupting mitochondrial membrane potential, and modulating processes such as autophagy and ferroptosis, etc. For example, a recent study reported that Prunin, a flavonoid, induces apoptosis through the PI3K/Akt/mTOR and MAPK pathways, contributing to its tumor-suppressive and antiproliferative effects [8]. Similarly, cedrol is a natural sesquiterpene alcohol reported to possess both in vitro and in vivo antiproliferative effects in colorectal cancer by inducing cell cycle arrest and caspase-dependent apoptotic cell death [9]. Kh has been reported to possess numerous bioactive phytochemicals. Therefore, its anticancer properties could be justified. Available reports on the bioactivity potential of the plant show that the plant has antiproliferative properties against cancer cells [7,10]. However, no report on the mechanism of action that underlies its anticancer properties has been reported. This study aims to explore the anticancer mechanism of action of the crude extract of Kh via cell-based assays, in addition to the exploration of anticancer phytochemicals responsible for the bioactivity.

## 2. Results

### 2.1. Bioactivity and Antioxidant Potentials of Kh

**Figure 1:**
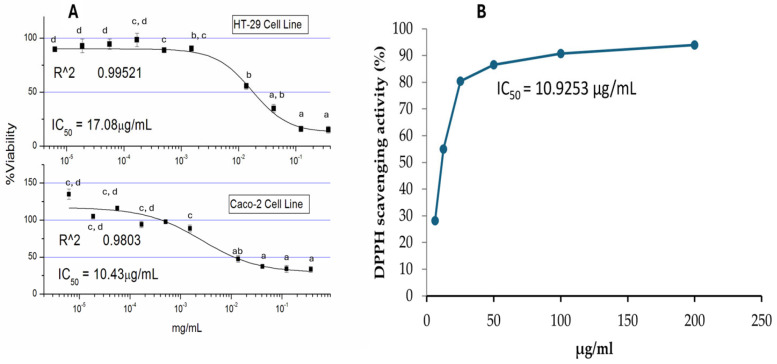
Bioactivity potentials of the crude methanolic extract of Kh against *Caco-2* cells. (**A**) represents the %viability curve, showing the IC50 value obtained from a triplicate of MTT tests. The %viability data are presented as mean ± standard deviation. ^a,b,c,d^ are the means within the same column, distinguished by different superscripts and exhibiting significant differences (*p* < 0.05). (**B**) represents the antioxidant potential of Kh via DPPH.

The bioactivity as say was conducted via MTT and DPPH antioxidant assays. The plant crude extract reduced the viability of colon carcinoma cell lines (*Caco-2* and HT-29 cells) at a low-microgram concentration with IC50 values of 10 µg/mL and 17 µg/mL, respectively (Figure 1A). Similarly, the plant crude extract shows a high antioxidant potential via the DPPH assay. Figure 1B shows the IC50 value of crude Kh at a low-microgram concentration.

### 2.2. Kh Induced Dual Apoptotic and Autophagic Flux Degradation Inhibition in Caco-2 Cells

**Figure 2:**
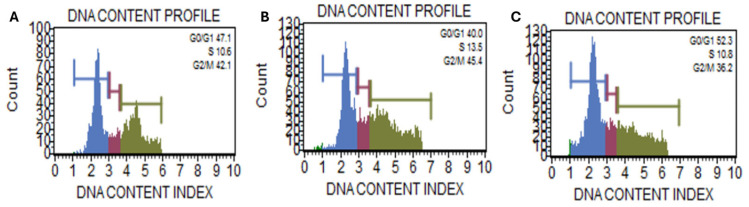
Cell cycle fraction analysis of the effect of Kh against *Caco-2* cells. (**A**) = vehicular control; (**B**) = treatment at 50 µg/mL and (**C**) = treatment at 100 µg/mL.

The cell cycle fraction analysis demonstrates a dose-dependent arrest of *Caco-2* cells at the G1 phase with increasing concentrations of Kh extract (Figure 2). At lower concentrations, the normal transition of cells through the cell cycle was evidenced. The increase in concentration at 100 µg/mL resulted in a shift from the G2/M phase to G0/G1 arrest. This is indicative that Kh may inhibit progression through the G2/M checkpoint, potentially by modulating key regulators such as cyclin B1 and Cdk1, which are essential for G2/M transition. In addition, the G0/G1 arrest could be due to the damaged cellular components supposed to be removed via autophagy. Reports have established autophagy as a cellular process that promotes cell survival by facilitating the removal of damaged proteins during cell cycle transitions, especially during stress. The failure of autophagy flux degradation is integral to cell cycle arrest and consequent senescence or apoptotic induction.

**Figure 3:**
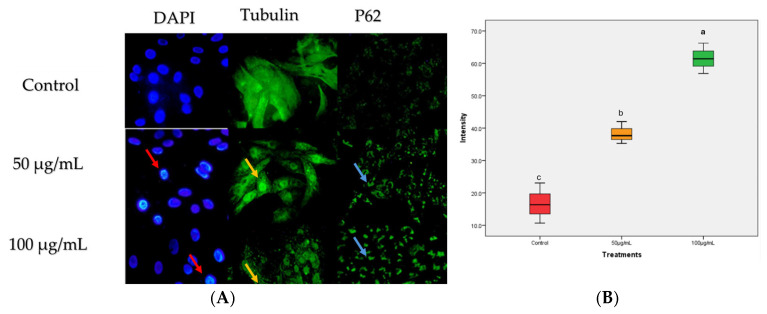
Photomicrographs of *Caco-2* cells treated with Kh at 50 and 100 µg/mL for 24 h. (**A**) The nuclei of treated cells at both concentration levels show nuclear fragmentation compared to the control. The immunostaining with β-tubulin shows a concentration-dependent degradation effect, while a concentration-dependent increase in the p62 puncta was observed compared with the control. All images were taken at 40× magnification. (**B**) Box plot displaying the concentration-dependent increment in the p62 intensity. The intensity data are presented as mean ± standard deviation. ^a,b,c^ are the means within the same column, distinguished by different superscripts and exhibiting significant differences (*p* < 0.05).

Further examination of the mechanism of action via cellular phenotyping assay with immunofluorescence assay shows that nuclei of the treated cells show signs of DNA fragmentation compared to the control (red arrows in Figure 3). A dose-dependent degradation of tubulin was also observed (yellow arrows in Figure 3), and conversely, p62 (known for autophagosome degradation) shows increased autophagosome puncta at both concentration levels (blue arrows in Figure 3). p62 is a selective autophagy receptor, and its degradation occurs during active autophagy induction. The increased p62 puncta suggest inhibition of autophagic flux degradation.

**Figure 4:**
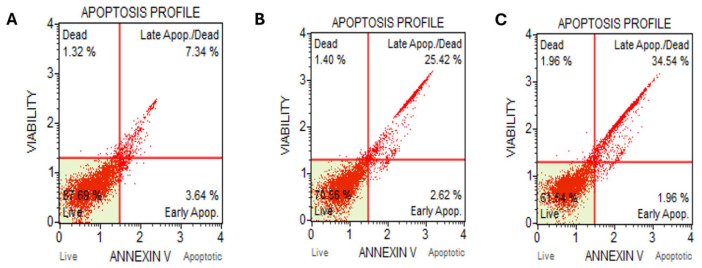
Apoptosis induction of crude methanolic extract of Kh against *Caco-2* cells. (**A**) Vehicle control; (**B**) treated with 50 µg/mL and (**C**) treated with 100 µg/mL.

Interestingly, a dose-dependent increase in late apoptosis was detected via Annexin V-FITC staining (Figure 4). Corroborating the earlier data of cell cycle fraction analysis arrest with autophagy flux degradation inhibition suggests the extract induced increased cellular stress that caused the shifting of earlier G2/M arrest observed at a lower concentration level to the G1 arrest at a higher concentration level and consequently cell cycle exit and apoptosis induction.

### 2.3. Kh Phytochemical Profile

**Figure 5:**
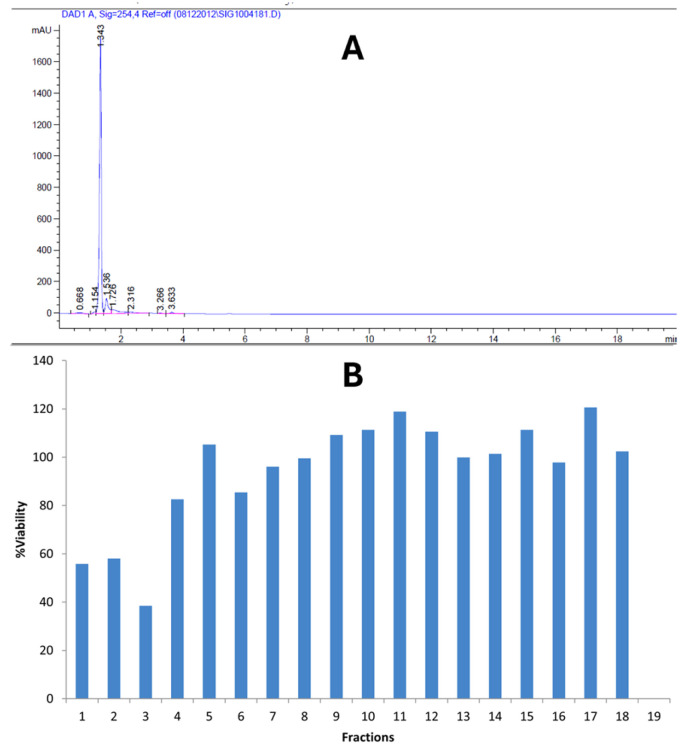
Bioactivity-guided fractionation of Kh executed via a gradient HPLC fractionation and MTT assay. (**A**) HPLC chromatograph; (**B**) bioactivity potential of all fractions using MTT assay.

The bioactivity-guided fractionation yielded some bioactive fractions against *Caco-2* cells. Figure 5A shows the chromatograph obtained from the HPLC fractionation of crude Kh, while the bioactivity potential of these fractions is shown in Figure 5B. Fractions 1–3 demonstrate some bioactivity potential and thus were pulled together for GCMS analysis.

**Figure 6:**
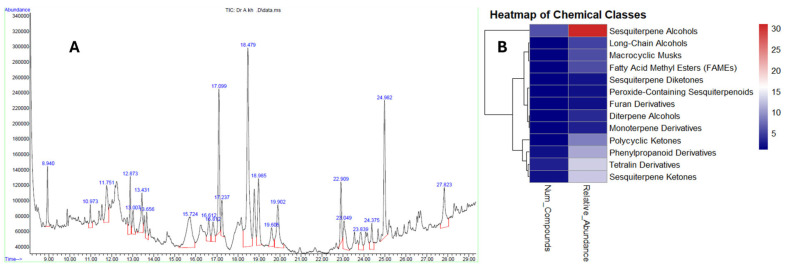
GCMS identification of anticancer phytochemicals present in Kh. (**A**) Chromatograph showing the peaks of the phytochemicals. (**B**) Summary of the number of phytochemicals grouped according to their respective chemical classes and their percentage abundance. Heatmap generated via R programming software (RStudio 2024.12.1 +563).

Fractions with growth inhibition at around 50% were pooled together for GCMS identification. Table 1 shows the phytochemical profiles present in fractions 1–3. Twenty-two (22) compounds belonging to various chemical classes were identified, including sesquiterpenoids as most abundant, phenylpropanoid derivatives, furan derivatives, long-chain (fatty) alcohols, fatty acid methyl esters, tetralin derivatives, and macrocyclic musks. Figure 6A shows the chromatographs showing the peaks obtained from GCMS, while Figure 6B displays the heatmap for the summary of the phytochemical classes, the number of phytochemicals in each class, and their relative abundance.

## 3. Discussion

In the ethnomedicinal herbal concoction, various medicinal plants are locally used as parts of the regimen for the treatment of ailments. The lack of scientific studies elucidating the mechanism of action of these plants remains a significant obstacle to the acceptance of many of these products. Kh is one of the plants that is usually present as a major ingredient in herbal concoctions among the people from the southwestern part of Nigeria. This plant has been reported to possess bioactive compounds including phenols and limoniids, which have enabled its potential as enhancers of antioxidant enzymes and anti-inflammatory and anticancer effects [7,10]. In this study, Kh demonstrates potent antiproliferative and antioxidant properties. The elucidation of the anticancer mechanism in colon carcinoma demonstrates a potential dual cell death mechanism. In studies, natural products with strong antioxidant capacity can induce autophagosome accumulation without degradation, suggesting their blockage of the late stages of autophagy [11,12]. The accumulation of p62 puncta (Figure 3) suggests a degradation block of autophagosome flux. This accumulation of p62 indicates impaired autophagosome clearance, which potentially may be due to lysosomal dysfunction [13]. Autophagy impairment can be related to cellular stress, particularly mitochondrial damage, which can trigger a shift from a protective autophagic response to apoptotic cell death [14].

Under normal physiological conditions, the induction of autophagy can be a survival mechanism, i.e., removing damaged cellular organelles and reducing intracellular stress [15]. However, when autophagy is impaired, mitochondrial dysfunction may lead to cytochrome c release and caspase activation, driving apoptosis [16,17]. Interestingly, the cell cycle fraction analysis shows an increase in cell population in G1 (Figure 2). Therefore, this arrest later leads to apoptosis, as evidenced by the Annexin V assay. The p62 accumulation, along with the elevation of late apoptotic cells, suggests a failure in autophagosome degradation that results in apoptosis induction, aligning with previous reports on autophagy-apoptosis crosstalk [18]. This suggests Kh may impair autophagy as part of its cytotoxic mechanism in cancer cells and later switch to apoptosis.

The crosstalk between autophagy and apoptosis has been linked to shared molecular mediators [19]. For instance, the Bcl-2 family is influential in determining the fate of cells under stress [20]. Typically, whether the cell will undergo programmed cell death via apoptosis or autophagy depends on the cellular context and stress level. Bcl-2 is known for its involvement in the inhibition of the permeabilization of the mitochondrion outer membrane, preventing the release of cytochrome C and consequent apoptosis blockage [21]. Similarly, Bcl-2 can bind to Beclin-1 via its BH3 domain to inhibit autophagy [22]. However, under cellular stress, Bcl-2 can suppress both apoptosis and autophagy, but its exact effect depends on the balance of pro- and anti-apoptotic signals [23]. The dose-dependent increase in the accumulation of p62 puncta observed in treated *Caco-2* cells suggests impaired autophagic flux and downstream activation of pro-apoptosis signals. This molecular interplay supports the idea that the bioactive compounds in Kh may induce cell death by switching the balance between protective autophagy and programmed cell death, a mechanism relevant in anticancer strategies.

Phytochemical constituents of the bioactive fractions showed the abundance of some chemical classes known to be inducers of many cell death mechanisms. For instance, the GCMS analysis yielded six sesquiterpene alcohols, accounting for 30% of the total abundance (Figure 6A). Sesquiterpene alcohols belong to the C15 isoprenoid compounds with lipophilic terpene backbone and hydrophilic hydroxyl moieties. Their structure–activity relationship (SAR) facilitates their cellular uptakes and multi-targeted biological effects [24,25]. For example, Terpinen-4-ol is a sesquiterpene found as a component of the essential oil of many plants. It was reported to affect cellular pathways, such as the modulation of Bcl-2 anti-apoptotic protein, and the induction of autophagy-related protein in human leukemia cells [26]. A sesquiterpene alcohol Costunolide isolated from *Michelia compressa* has been reported to disrupt microtubule dynamics by directly interacting with tubulin subunits in MCF7 cells [27]. Interestingly, 1,2-Longidione (a sesquiterpene diketone) and Ambrox (a derivative of ambrein) reported as constituents of Kh have structures that suggest interactions with microtubule dynamics, potentially leading to tubulin degradation and cell cycle arrest [28]. Sesquiterpene alcohols are known to be inducers of ROS-mediated DNA damage, late apoptosis, autophagy flux inhibition, and G1-phase arrest [29,30,31]. Together, these mechanisms underscore the potential of sesquiterpene alcohols as structurally driven, multi-target anticancer agents with promising therapeutic relevance.

Furthermore, previous reports have observed the presence of Limnoids in Kh. Our result shows the presence of furan derivatives, closely related to the Limnoids. Several other phytochemicals, such as 2-Propenoic acid, 3-(2,4-dimethoxyphenyl)-, (E)-, and a phenylpropanoid derivative, have been reported for their antioxidant properties and apoptosis induction in cancer cells. Meanwhile, there are limited reports on these specific compounds. Their similarities to known anticancer agents warrant further investigation into their mechanisms of action, including their effects on autophagy, apoptosis, and cell cycle regulation.

## 4. Materials and Methods

### 4.1. Plant Extraction

The bark of the plant Kh was purchased from a local herbal vendor in Iwo Township, Osun state, Nigeria. A voucher specimen with Voucher No. UIH-23570 was deposited at the Herbarium of the University of Ibadan, accessible for reference. The plant part was ground to powder form and extracted via maceration in HPLC-grade methanol (obtained from Sigma St. Louis, MO, USA) for 24 h in a 10% weight-to-volume ratio, under continuous shaking at room temperature. The crude extract was filtered out, and the filtrate was concentrated in a rotary evaporator. The crude extract was reconstituted in methanol at 10 mg/mL and stored at −20 °C for further experiments.

### 4.2. DPPH Assay

The DPPH scavenging activity of Kh was examined using the DPPH assay method, as described by [32]. Briefly, 0.1 mmol L^−1^ DPPH was prepared in methanol. Thereafter, 0.75 mL of Kh was added to the DPPH solution. The mixture was left at room temperature for 20 min, and the absorbance was recorded at 517 nm. The percentage of the DPPH scavenging activity of the extract was estimated using the following formula:

DPPH scavenging activity (%) = [(absorbance of control − absorbance of sample extract)/absorbance control] × 100

where absorbance of control is the absorbance of methanol + DPPH, and absorbance of sample is the absorbance of DPPH radical + sample extract.

### 4.3. Bioactivity Test

Colon carcinoma cells (Caco-2 ATCC collection: Caco-2: HTB-37 and HT-29: HTB-38) were maintained under a standard cell culture protocol. Around 90% of confluence cells were passaged in 10% FBS DMEM media and cultured in a 5% CO_2_ incubator at 37 °C. MTT assay was performed to determine the antiproliferative potential of the methanol crude extract. A total of 120 µL of 50,000 cells/mL was seeded in 96-well plates and treated with serially diluted crude extract. The plate was allowed incubation for 72 h in a 5% CO_2_ incubator at 37 °C. MTT salt was later added to the culture, and the formed formazan was dissolved in isopropanol. Optical density was measured with a plate reader, and IC50 of the crude extract against the cancer cell line was calculated using Origin 6.1 software (OriginLab Corporation, Northampton, MA, USA).

### 4.4. Cellular Phenotyping Assay

Microscopy techniques such as light and immunofluorescence microscopy were used to examine the phenotypic changes in the cellular architecture that are integral to the cell death mechanism. Typically, immunofluorescence staining was performed for 24 h. Post treatment of *Caco-2* cells with the crude extract, the cells were fixed in 1:1 Methanol: Acetone and 3.75% Formaldehyde in PBS for tubulin and p62, respectively. Cells were washed, and primary antibodies (β tubulin: mouse monoclonal; p62: rabbit monoclonal) were prepared in 5% FBS in PBS and incubated at 37 °C for 2 h. Alexa 488 conjugated secondary antibodies (goat anti-mouse and goat anti-rabbit, respectively) were used in conjunction with the primary antibodies. The cells were counterstained by dropping anti-fade equipped with DAPI. Slides were scored using Nikon Eclipse Ci fluorescence microscope equipped with a NIS-Elements camera system (Nikon Corporation, Tokyo, Japan).

### 4.5. Cell Cycle Fraction Analysis

*Caco-2* cells at around 75% confluence were treated with the plant extract at 50 µg/mL and 100 µg/mL, respectively, and methanol served as vehicular control. After 24 h. of drug incubation, the cells were harvested by centrifugation and fixed in 1:1 ice-cold PBS and ethanol. Thereafter, the cells were washed with PBS and then treated with 0.1% *w*/*v* saponin and RNAse, and the nuclei were stained with propidium iodide for 30 min at 37 °C. Cell cycle fractions were analyzed with a flow cytometer FACS Muse cell analyzer (Sigma, St. Louis, MO, USA).

### 4.6. Annexin V FITC Assay

Measurement of apoptosis in the treated and untreated *Caco-2* cells was performed using an Annexin V FITS apoptosis detection kit from Abcam (ab14085). Briefly, the treatment of cells at 50 µg/mL and 100 µg/mL and vehicular control were harvested 24 h after treatment. The cells were washed with PBS and stained with fluorescein-conjugated Annexin V FITC and propidium iodide for 30 min at room temperature in the dark. Following incubation, 500 μL annexin V binding buffer was added to the cells, and percentage apoptosis was measured using a Flow cytometer FACS Muse cell analyzer (Sigma, St. Louis, MO, USA).

### 4.7. Phytochemical Analysis

#### 4.7.1. HPLC Separation

The chemical investigation and profiling of potential anticancer phytochemicals present in the Kh crude extract was conducted using a bioactivity-guided fractionation approach. A gradient HPLC fractionation was performed using HPLC analysis with an Agilent Technologies 1290 Infinity system (Agilent Inc., Palo Alto, CA, USA). A total of 5 µL of the crude extract was automatically injected into the HPLC ZOBRAX RX-C18 column (4.6 × 150 mm) as a stationary phase, and the mobile phases consisted of A: 0.1% acetic acid in 90% acetonitrile and B: 0.1% acetic acid in 90% water. The column temperature was maintained at 27 °C. The chromatogram was recorded at 254 nm. The gradient separation yielded 20 fractions obtained at 1mL/min flow rate time. The resulting fractions were dried, reconstituted in methanol, and filtered through a 0.22 μm syringe filter prior to GC-MS analysis, and a part of them was used in a bioactivity test via MTT assay. The fractions that show growth inhibition around 50% in *Caco-2* cells were pooled together for GC-MS analysis. No derivatization was performed before the injection.

#### 4.7.2. GCMS Identification

The bioactive fractions that possessed growth inhibition of *Caco-2* cells at around 50% inhibition were pooled together and further analyzed for the identification of phytochemicals. In accordance with the data obtained, the first three fractions were pooled together in the bioactivity test and the HPLC chromatograph, and GCMS analysis was performed. Essentially, 2 µL was injected via the auto-sampler injection system of the GC-MS 7890B GC system from Agilent Technologies (Santa Clara, CA, USA). The products were identified using the database-integrated software (NIST MS (NIST-08 MS library, Gaithersburg, MD, USA)). The identification of the sample components was achieved using gas chromatography coupled with a mass-selective detector (GC-MS). For the separation of target compounds, a DB-5 MS capillary column from Agilent technologies (30 m length × 0.25 mm internal diameter, phase thickness 0.25 μm) was used with helium as the carrier gas at a flow rate of 1mL/min, inlet temperature 250 °C with a split mode ratio (50), and oven temperature ranging from 50 to 250 °C with a total analysis time of 61 min. The MS detector was set as follows: acquisition scan type, mass ranging from 40 to 500 g/mol, scan speed 1.56, 4 min solvent delay, and 230 °C MS source temperature.

## 5. Statistical Analysis

The data generated from this study were statistically analyzed using SPSS (version 20). One-way analysis of variance (ANOVA) was performed to separate the means and significance level determination at (*p* < 0.05). ^a,b,c,d^ mean significant differences within the same column (*p* < 0.05).

## 6. Conclusions

This study demonstrated the anticancer potential of the methanol crude extract of Kh, primarily through the modulation of the autophagy–apoptosis pathways. The observed inhibition of autophagic flux, coupled with increased apoptotic cell death, suggests that the extract may sensitize cancer cells to apoptosis by impairing the clearance of autophagosomes. This mechanism is particularly relevant in cancers that depend on autophagy for survival and resistance to therapy. However, a key limitation of this study is the use of crude extract rather than isolated pure compounds. As such, the specific bioactive constituent(s) responsible for the observed effects remain unidentified. Future research should focus on the isolation and structural characterization of individual anticancer phytochemicals from Kh, along with detailed in vivo mechanistic studies to further elucidate the interplay between autophagy and apoptosis. In addition, lysosomal dysfunction and immunoblotting assays are recommended for further exploration of autophagy and apoptosis crosstalk. A deeper understanding of this crosstalk could provide valuable insights for the development of autophagy-targeted therapies in cancer treatment.

## Figures and Tables

**Table 1:** GCMS profiling of the possible anticancer phytochemicals present in Kh.

Peak	Rt (min)	Compound Name	Chemical Class	Area (%)	MF	Mw
1	8.94	7-epi-cis-Sesquisabinene hydrate	Sesquiterpene alcohol	2.5593	C_15_H_26_O	222
2	10.973	Z,Z-2,5-Pentadecadien-1-ol	Long-chain alcohol	1.356	C_15_H_28_O	224
3	11.751	13-nor-Eremophil-1(10)-en-11-one	Sesquiterpene ketone	4.2434	C_14_H_22_O	206
4	12.873	2,5,5,8a-Tetramethyl-3,5,6,7,8,8a-hexahydro-2H-naphthalen-1-one	Sesquiterpene ketone	3.1604	C_14_H_22_O	206
5	13.007	Edulan II	Furan derivative	1.6017	C_13_H_20_O	192
6	13.431	2-Butenal, 2-methyl-4-(2,6,6-trimethyl-1-cyclohexen-1-yl)-	Monoterpene derivative	3.081	C_14_H_22_O	206
7	13.656	Ethanone, 1-(5,6,7,8-tetrahydro-2,8,8-trimethyl-4H-cyclohepta[b]furan-5-yl)-	Sesquiterpene ketone	1.6636	C_14_H_20_O_2_	220
8	15.724	2-Propenoic acid, 3(2,4-dimethoxyphenyl)-, (E)-	Phenylpropanoid derivative	8.2974	C_11_H_12_O_4_	208
9	16.612	Cedrol	Sesquiterpene alcohol	2.126	C_15_H_26_O	222
10	16.812	2-Acetoxy-1,1,10-trimethyl-6,9-epidioxydecalin	Peroxide-containing sesquiterpenoid	2.0345	C_15_H_24_O_4_	268
11	17.099	Cedrane, 8-propoxy-	Sesquiterpene ether	8.6444	C_18_H_32_O	264
12	17.237	Z, E-2,13-Octadecadien-1-ol	Long-chain alcohol	2.2318	C_18_H_34_O	266
13	18.479	(7a-Isopropenyl-4,5-dimethyloctahydroinden-4-yl) methanol	Sesquiterpene alcohol	20.5561	C_15_H_26_O	222
14	18.985	Patchouli alcohol	Sesquiterpene alcohol	5.6829	C_15_H_26_O	222
15	19.605	1,2-Longidione	Sesquiterpene diketone	2.1346	C_15_H_22_O_2_	234
16	19.902	3a,9-Dimethyldodecahydrocyclohepta[d]inden-3-one	Polycyclic ketone	5.6674	C_16_H_26_O	234
17	22.909	Ambrox	Diterpene alcohol	3.4322	C_16_H_28_O	236
18	23.049	1-(3-Hydroxypropyl) -5,5,8a-trimethyldecahydronaphthalen-2-ol	Sesquiterpene alcohol	3.189	C_16_H_30_O_2_	254
19	23.839	4,7,10-Hexadecatrienoic acid, methyl ester	Fatty acid methyl ester	2.0859	C_17_H_28_O_2_	264
20	24.375	5,8,11,14-Eicosatetraenoic acid, methyl ester, (all-Z)-	Fatty acid methyl ester	2.1711	C_21_H_34_O_2_	318
21	24.982	7-Acetyl-6-ethyl-1,1,4,4-tetramethyltetralin	Tetralin derivative	9.167	C_18_H_26_O	258
22	27.823	Ethylene brassylate	Macrocyclic musk	4.9144	C_15_H_26_O_4_	270

## Data Availability

Data are contained in the article.

## Abbreviations

Kh	*Khaya grandiofoliola*
DPPH	2,2-diphenyl-1-picrylhydrazyl
MTT	3-(4,5-dimethylthiazol-2-yl)-2,5-diphenyltetrazolium bromide
HPLC	High-performance liquid chromatography
GCMS	Gas chromatography mass spectrometry
